# Simultaneous quantitative screening of 53 phytochemicals from *Rheum tataricum* L. roots: a comparative study of supercritical CO_2_, subcritical ethanol, and ultrasound-assisted extraction for enhanced antioxidant, antibacterial activities, and molecular docking study

**DOI:** 10.3389/fpls.2024.1513875

**Published:** 2024-12-06

**Authors:** Madina Amangeldinova, Mehmet Ersatır, Adem Necip, Mustafa Abdullah Yilmaz, Mehmet Cimentepe, Nataliya Kudrina, Nina V. Terletskaya, Ozge Ozturk Cimentepe, Metin Yildirim

**Affiliations:** ^1^ Faculty of Biology and Biotechnology, Al-Farabi Kazakh National University, Almaty, Kazakhstan; ^2^ Institute of Genetics and Physiology, Almaty, Kazakhstan; ^3^ Department of Chemistry, Faculty of Art and Science, Cukurova University, Adana, Türkiye; ^4^ Department of Pharmacy Services, Vocational School of Health Services, Harran University, Sanliurfa, Türkiye; ^5^ Dicle University Science and Technology Research and Application Center, Diyarbakir, Türkiye; ^6^ Department of Analytical Chemistry, Dicle University, Faculty of Pharmacy, Diyarbakir, Türkiye; ^7^ Department of Pharmaceutical Microbiology, Faculty of Pharmacy, Harran University, Sanliurfa, Türkiye; ^8^ Department of Pharmacology, Faculty of Pharmacy, Harran University, Sanliurfa, Türkiye; ^9^ Department of Biochemistry, Faculty of Pharmacy, Harran University, Sanliurfa, Türkiye

**Keywords:** *Rheum tatarium* L., supercritical carbon dioxide, subcritical ethanol, ultrasound assisted extraction, biological activities

## Abstract

In this study, *Rheum tataricum* L. extracts were obtained using various green extraction techniques, including supercritical CO_2_, subcritical ethanol, and ultrasound-assisted extraction, each performed under optimized parameters. The phytochemical content of the extracts was analyzed using the LC-MS/MS technique, quantifying 53 phytochemicals. Additionally, the *in vitro* antioxidant properties and antibacterial activities of the extracts were evaluated against *Staphylococcus aureus* and *Enterococcus faecalis* as gram-positive bacteria, and *Escherichia coli* and *Pseudomonas aerugino*sa as gram-negative bacteria. According to the results, the extracts were rich in catechin, epicatechin, cyranoside, and chlorogenic acid. Extracts obtained via ultrasonic extraction demonstrated stronger antioxidant properties. The IC_50_ values for the DPPH radical scavenging activity of obtained extracts ranged between 0.0173 mg/mL and 0.0400 mg/mL. The highest total phenolic content was found in the UAE-M-4h extract (213.44 mg GAE/mL). The extracts prepared with UAE-MeOH-2h-4h, UAE-EtOH-2h-4h, Sbc-EtOH-E-140-60-80, Sc-90 atm, and Sc-400 atm showed antibacterial activity against both Gram-positive and Gram-negative bacteria at varying rates (MIC range: 31.25 to 250 μg/mL). Based on the all results, the ultrasound assisted extraction proved superior to the other techniques. This study, utilizing three different extraction methods with varying variables such as temperature, pressure, and extraction time, has provided significant insights into which extraction method should be employed for isolating specific phytochemicals or for therapeutic purposes, based on the differing antibacterial results observed. The findings highlight the importance of selecting the appropriate extraction method depending on the target phytochemical or desired antibacterial effect in treatment applications.

## 1 Introduction

Tatar rhubarb (*Rheum tataricum* L.) is a widespread plant in the Republic of Kazakhstan, classified as an ephemeral species (Gemejiyeva and Grudzinskaya, 2018). Its distribution area includes plains, desert regions, and industrial zones in Russia, Xinjiang, and Afghanistan, where it is useful as a raw material source. The study of wild rhubarb species, such as *R. tataricum*, presents a significant scientific challenge due to their limited habitats within narrow strips of dry steppes and deserts in Central Asia, stretching from the northeast of the Astrakhan region in Russia to Lake Balkhash in Kazakhstan (Гемеджиевa et al., 2017; Golubkina et al., 2022).

In recent years, rhubarb has become an increasingly promising research object due to its unique nutritional properties, high antioxidant levels, and wide range of applications in medicine and agriculture (Jiao and Du, 2000; Yildirim et al., 2021). It serves as a possible source of raw materials for creating herbal remedies with various properties, including anti-inflammatory, astringent, laxative, hemostatic, antitumor, and other properties (2014). Therefore, many researchers pay great attention to the use of *Rheum tataricum* for medical and food purposes (Dai et al., 2015; Shahrajabian et al., 2022). This plant contains anthocyanins and their derivatives, including cyanidin-3-glucoside, cyanidin-3-rutinoside, chrysanthemin, and cyanine (Agarwal et al., 2001).

Such secondary metabolites tend to decompose under the influence of various factors, such as temperature, pressure, and the solvent used to extract plant materials.

It should be noted that the method and conditions of extraction of biologically active compounds play an essential role in their wide application in medicine and pharmacology (Yıldırım et al., 2024). Numerous traditional extraction methods exist, such as hydrodistillation, which is often used for extracting essential oils. For the extraction of biologically active compounds (BACs) from plant materials, maceration has been traditionally employed, during which solvents are added in combination with heating or stirring to enhance the solubility of secondary metabolites (Ćujić et al., 2016; Cacique et al., 2020). Over the past decades, intensive research aimed at improving extraction methods has been driven by the growing public interest in using natural compounds and the increased awareness of the need for sustainable development and environmental protection (Picot-Allain et al., 2021). Traditional methods, such as percolation with organic solvents, Soxhlet extraction, rectification, and infusion, have certain drawbacks, including the use of hazardous solvents, lengthy processes, high resource consumption, and low efficiency in extracting secondary metabolites (Kubátová et al., 2001; Yıldırım et al., 2025b).

In order to improve the efficiency of the processes and enhance safety while maintaining high quality, various types of new green extraction methods have been developed such as ultrasound-assisted extraction (UAE), microwave-assisted extraction, supercritical CO_2_ extraction and subcritical ethanol (sbcEtOH-E) (Chemat et al., 2012; Demirkol et al., 2022). Green extraction methods should fully comply with sustainable development strategies and the six principles of green extraction, such as using alternative solvents, reducing energy consumption, and striving for biodegradable extract (Li et al., 2004).

SC-CO_2_ is one of the techniques developed from SFE technology. This method refers to the “green strategy” for efficiently extracting valuable chemical compounds from various plant materials by controlling the fluid density by changing the pressure and, less commonly, temperature (Demirkol et al., 2022).

UAE, known as a green method, involves the disruption of plant cell walls through the application of ultrasonic waves, resulting in the release of active compounds. It is relatively simple to use and does not require significant investment (Fu et al., 2021). Ultrasound can extract all known compounds produced by plants from plant raw materials, and it is not limited by the polarity or molecular weight of the component, making it suitable for extracting a wide range of BACs. In this method, ultrasound generates sound waves that create laminar or turbulent flow, enhancing the efficiency of the extraction process for biologically active substances (Rahaman et al., 2019; Hadidi et al., 2020; Qin et al., 2021). It is worth noting that water serves as the primary medium for the propagation of ultrasonic waves during ultrasonic-assisted extraction (Panadare et al., 2020). Solvents commonly used in UAE include ethanol (for extracting phenols, aldehydes, and esters), chloroform (for extracting fatty acids, spices, and other fat-soluble substances), ether, and benzene (for extracting phenols and other aromatic compounds). When selecting an extraction solvent, it is important to adhere to the principles of green extraction and consider factors such as environmental safety, mass transfer, toxicity to the organism, financial feasibility, and the type of biologically active compound. For instance, in studies by Bellumori et al. (2016), it was demonstrated that the use of ethanol in the extraction of rosmarinic and carnosic acids from rosemary leaves resulted in a high yield of rosmarinic acid (6.8%), while the use of n-hexane as a solvent led to high yields of carnosic acids (13%) (Bellumori et al., 2016). Moreover, utilizing UAE significantly reduces the total solvent volume needed for the extraction process (Chemat et al., 2017).

sbcEtOH-E is considered a relatively new and green extraction method that does not require alternative energy sources, such as microwaves or ultrasound. Compared to subcritical water extraction, sbcEtOH-E does not require high temperatures to reach a subcritical state, which can be unsafe for thermolabile compounds (Wang et al., 2017; Marcus, 2018).

According to the literature, no studies have shown a comparative optimization of parameters for the best yield of biologically active compounds from the roots of *Rheum tataricum* L. This study aims to determine the optimal conditions for the extraction of biologically active compounds from the roots of *Rheum tataricum* L. using green extraction methods such as SC-CO_2_, sbcEtOH-E, and UAE. Additionally, it seeks to analyze the composition of extracts prepared under different parameters using these methods through LC-MS/MS analysis, and to evaluate their *in silico*, antioxidant and antibacterial properties.

## 2 Materials and method

### 2.1 Plant material

The object of the study is *Rheum tataricum* L., collected on April 20, 2024, in the Sjugatinskaya Valley, located between the Charyn and Chilik rivers (coordinates: 43° 26′ 00″ N, 78° 59′ 00″ E). The collected *Rheum tataricum* L. sample was verified at the “Institute of Botany and Phytointroduction” in Almaty, Kazakhstan. The samples were dried in vacuum drying at 45-50°C and ground into powder. The dried plant material is stored at Harran University (Şanlıurfa, Turkey).

### 2.2 Extraction methods

#### 2.2.1 sbcEtOH-E extraction

The experiments were conducted using a device designed for sub-critical solvent extraction. sbcEtOH-E was performed using 1 g of *Rheum tataricum* L. roots at a temperature of 140°C, a pressure of 60 and 80 atm, with a 30-minute static extraction time followed by a 20-minute dynamic extraction using ethyl alcohol at a flow rate of 2 mL per minute. All extractions were done in triplicate.

#### 2.2.2 ScCO_2_ extraction

*Rheum tataricum* L. roots extracts were prepared using a supercritical CO_2_ extractor (Supercritical Extraction System SuperEx F series 500, Türkiye). Briefly, 25 grams of plant root (in a polyester pouch) was placed into the extractor vessel. The temperature values for the extractor, restrictor, and separator were set to 60°C, 120°C, and 60°C, respectively. The pressure was set to 90 and 400 atm. All extractions were performed at least three times.

#### 2.2.3 UAE extraction

Ultrasound assisted extraction experiments of *Rheum tataricum* L. roots were performed using Elmasonic Select 150 device (19.9 in. x 11.8 in. surface area and 3.9 in. depth). For each experiment, 2 g of plant root and 30 ml of solvent were combined in a 50-ml capped sample tube. Experiments were performed at room temperature using two different solvents [methanol (MeOH) and ethanol (EtOH)] with two different extraction times (1 h and 4 h). All extractions were performed at least three times.

### 2.3 Mass spectrometer and chromatograph conditions for LC-MS/MS analysis

A Shimadzu-Nexera model ultrahigh performance liquid chromatograph (UHPLC) coupled with a tandem mass spectrometer was used for the identification of phytochemicals, and the detailed protocol is provided in the **Supplementary Materials** (Yilmaz, 2020).

#### 2.3.1 Method validation studies for LC–MS/MS

The method validation study for LC/MSMS applied to the extracts of *Rheum tataricum* L. roots obtained under various extraction methods were conducted according to the procedure in the literature (Gemejiyeva and Grudzinskaya, 2018). The parameters related to the LC–MS/MS method validation studies given with table at **Supplementary Materials**.

### 2.4 Antioxidant activity

#### 2.4.1 DPPH radical scavenging activity method

The DPPH free radical scavenging activity was determined using the DPPH˙ (1,1-diphenyl-2-picrylhydrazyl) method. Plant extracts were prepared at a concentration of 1.00 mg/mL. In this study, Trolox, BHA, and BHT were used as standard solutions at different concentrations. To 1 mL of an ethanol-water mixture, 0.5 mL of DPPH radical solution was added and vortexed, then incubated in the dark for half an hour. The absorbance was measured at 517 nm using a UV-VIS spectrophotometer. Results were expressed as IC_50_ (mg/mL) (Necip and Işık, 2019).

#### 2.4.2 ABTS radical scavenging activity method

The ABTS free radical scavenging activity was described in detail in our previous study. Briefly, prepared ABTS radicals were mixed with standard and sample solutions and incubated in the dark for half an hour. After incubation, the absorbance values of the samples were measured at 734 nm using a UV spectrophotometer. Results were expressed as IC_50_ (mg/mL) (Necip et al., 2021).

#### 2.4.3 Cu^2+^-Cu^+^ reducing activity

Plant extracts and Trolox at different concentrations were mixed with neocuproine (7.5 mM), NH4Ac (1 M), and 0.25 mL CuCl_2_ (0.01 M). The absorbance was measured at 450 nm using a UV-VIS spectrophotometer. The results were expressed as Trolox equivalent mg TE/mL.

#### 2.4.4 Determination of total phenolic content

A 50 µL aliquot of the plant extract was taken, and 1.00 mL of distilled water was added to each sample, followed by 25 µL of Folin-Ciocalteu reagent, ensuring homogeneity through mixing. After 3 minutes, 40 µL of 20% sodium carbonate was added to the prepared samples, which were then vortexed and incubated at room temperature in the dark for two hours. The absorbance values were measured at a wavelength of 760 nm using a UV spectrophotometer. The corresponding amount of gallic acid equivalent was calculated based on the measured absorbance. The results were expressed as mg GAE/mg extract (Necip and Durgun, 2022).

### 2.5 Antibacterial activity

The broth microdilution test was studied according to our previous study (Weinstein and Lewis, 2020). *Staphylococcus aureus* ATCC 29213, *Enterococcus faecalis* ATCC 29212 as gram positive bacteria, *Escherichia coli* ATCC 35150 and *Pseudomonas aeruginosa* ATCC 27853 as gram negative bacteria, were used in this study.

*Rheum tataricum L.* extracts (3.91 to 2000 μg/mL) were added in each well of 96-well microtiter plate. The bacterial suspension, containing approximately 5 × 10^6^ colony-forming units/mL, was incubated on plates at 37°C for 24 h. The absorbance values were determined using a microplate spectrophotometer (Thermo Fisher Scientific, USA) at 570 nm. The lowest concentration of *Rheum tataricum L. extract* that indicated no growth was determined as MIC. All studies were performed at least three times (Yildirim et al., 2025b). Minimum bactericidal concentration (MBC) was considered the lowest concentration of the *Rheum tataricum L.* different extract that results in the killing of 99.9% of the bacteria after the incubation period at 37°C for 24 h.

### 2.6 Molecular docking

The molecular docking studies were conducted based on our previous studies (Yildirim et al., 2025a; Yildirim et al., 2025b). Briefly, Molecular docking studies were carried out using the Maestro 13.8 Schrodinger ¨ 2023–3 program (https://www.schrodinger.com). Three dimensional crystal structures of the proteins were obtained from the RCSB Protein Data Bank (https://www.rcsb.org). Ligands and proteins were prepared using Wizard in the Maestro, Schrodinger packageCalculations were performed by docking the ligands to the proteins of *S. aureus*, *E. faecalis*, *P. aeruginosa*, and *E. coli*, using the proteins 1JIJ, 4WUB, 2UV0, and 6QXS, respectively. Docking studies were conducted using Schrödinger’s Glide/XP module.

### 2.7 Statistical analysis

All statistical analyses were implemented with GraphPad Prism 9 (GraphPad Software Inc., USA) with one-way analysis of variance (ANOVA) followed by Tukey’s multiple comparison test.

## 3 Results and discussion

### 3.1 Characterization

TIC (Total Ion Chromatogram) chromatogram of standard phenolic compounds analysed by the developed LC–MS/MS method. LC-MS/MS spectrums of *Rheum tataricum* L. root extracts obtained under different conditions are given in the **Supplementary Materials**.

**Figure 1:**
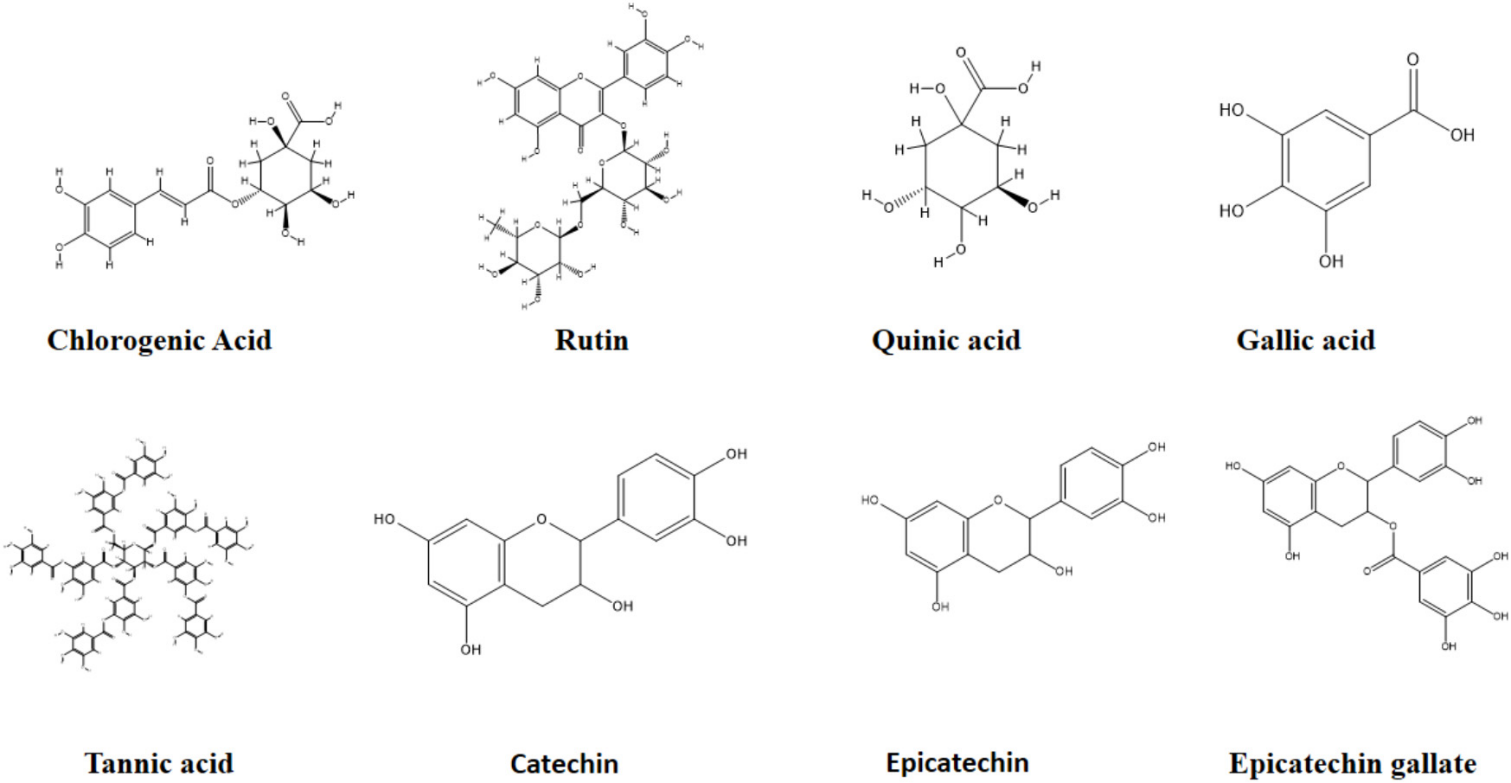
Representative compounds identified in *Rheum tataricum* L. extracts.

The LC-MS/MS results regarding the presence of 53 phytochemicals in the extracts of *Rheum tataricum* L. roots are presented in **Table 1**. As shown in the **Table 1**, although 13 phytochemicals, including important ones such as quinic acid, gallic acid, tannic acid, rutin, hesperidin, catechin, and naringenin, were found in all extracts, 31 phytochemicals were not detected in any of the extracts (**Figure 1**). Phytochemicals such as epicatechin, vanillic acid, daidzin, salicylic acid, luteolin, kaempferol, and isoquercitrin were found in some extracts depending on the extraction conditions, while they were not detected in others.

**Table 1:** The LC-MS/MS results regarding the presence of 53 phytochemicals in the extracts of *Rheum tataricum* L. roots.

No	Analytes	UAE-E-2h	UAE-E-4h	UAE-M-2h	UAE-M-4h	Sc-90 atm	Sc-400 atm	Sbc-EtOH-140-60	Sbc-EtOH-140-80
**1**	**Quinic acid**	0.708	0.21	0.347	0.426	0.501	0.075	0.654	0.182
**2**	**Epicatechin gallate**	0.826	1.182	1.135	1.07	†	†	0.998	1.193
**3**	**Aconitic acid**	†	†	†	†	†	†	†	†
**4**	**Syringic aldehyde**	†	†	†	†	†	†	†	†
**5**	**Epigallocatechin**	†	†	†	†	†	†	†	†
**6**	**Protocatechuic acid**	1.078	1.118	0.837	0.9	0.088	0.055	2.934	1.971
**7**	**Catechin**	7.641	8.712	7.812	7.483	0.266	0.225	5.297	6.032
**8**	**Gentisic acid**	†	†	†	†	†	†	†	†
**9**	**Chlorogenic acid**	13.749	14.019	12.67	12.412	2.906	2.983	10.266	10.585
**10**	**Protocatechuic aldehyde**	0.152	0.153	0.13	0.141	0.095	0.061	0.527	0.347
**11**	**Tannic acid**	0.062	0.066	0.077	0.081	0.037	0.02	0.083	0.065
**12**	**Epigallocatechin gallate**	†	†	†	†	†	†	†	†
**13**	**Cynarin**	†	†	†	†	†	†	†	†
**14**	**4-OH Benzoic acid**	0.435	0.629	0.503	0.545	0.142	0.137	0.805	0.646
**15**	**Epicatechin**	10.307	11.936	11.744	11.799	†	†	9.158	10.273
**16**	**Vanilic acid**	†	†	†	†	†	†	†	†
**17**	**Caffeic acid**	†	†	†	†	†	†	0.022	0.014
**18**	**Syringic acid**	†	†	†	†	†	†	†	†
**19**	**Sinapic acid**	†	†	†	†	†	†	†	†
**20**	**Daidzin**	0.033	0.038	0.037	0.039	†	†	0.026	0.029
**21**	**Piceid**	†	†	†	†	†	†	†	†
**22**	**p-Coumaric acid**	0.12	0.115	0.109	0.114	0.062	0.038	0.209	0.166
**23**	**Ferulic acid-D3-IS^h^**	*	*	*	*	*	*	*	*
**24**	**isoquercitrin**	0.08	0.088	0.065	0.084	†	†	0.11	0.1
**25**	**Ferulic acid**	†	†	†	†	†	†	†	†
**26**	**Acacetin**	†	†	†	†	†	†	†	†
**27**	**Vanillin**	†	†	†	†	†	†	†	†
**28**	**Genistin**	†	†	†	†	†	†	†	†
**29**	**Coumarin**	†	†	†	†	†	†	†	†
**30**	**Chrysin**	†	†	†	†	†	†	†	†
**31**	**Cyranoside**	4.045	4.332	3.851	3.823	0.435	0.453	3.923	3.745
**32**	**Miquelianin**	†	†	†	†	†	†	†	†
**33**	**Rutin-D3-IS**	*	*	*	*	*	*	*	*
**34**	**Fisetin**	†	†	†	†	†	†	†	†
**35**	**Hesperidin**	0.315	0.366	0.295	0.291	0.024	0.021	0.466	0.431
**36**	**o-Coumaric acid**	†	†	†	†	†	†	†	†
**37**	**Astragalin**	†	†	†	†	†	†	0.009	†
**38**	**Rosmarinic acid**	†	†	†	†	†	†	†	†
**39**	**Ellagic acid**	†	†	†	†	†	†	†	†
**40**	**Rutin**	0.765	0.928	0.723	0.789	0.044	0.04	1.116	1.047
**41**	**Quercitrin**	†	†	†	†	†	†	†	†
**42**	**Amentoflavone**	†	†	†	†	†	†	†	†
**43**	**Nicotiflorin**	0.069	0.086	0.073	0.074	†	†	0.116	0.085
**44**	**Salicylic acid**	†	†	†	†	†	†	†	†
**45**	**Gallic acid**	0.449	0.607	0.5	0.542	0.033	0.014	1.53	0.917
**46**	**Quercetin-D3-IS**	*	*	*	*	*	*	*	*
**47**	**Quercetin**	†	†	†	†	†	†	†	†
**48**	**Luteolin**	†	0.003	†	†	†	†	0.007	0.004
**49**	**Hesperetin**	†	†	†	†	†	†	†	†
**50**	**Naringenin**	0.073	0.079	0.07	0.067	0.065	0.033	0.087	0.082
**51**	**Genistein**	†	†	†	†	†	†	†	†
**52**	**Kaempferol**	0.011	0.008	0.009	0.006	†	†	0.021	0.007
**53**	**Apigenin**	†	†	†	†	†	†	†	†
**54**	**Fumaric acid**	†	†	†	†	†	†	†	†
**55**	**Cosmosiin**	†	†	†	†	†	†	†	†
**56**	**Daidzein**	†	†	†	†	†	†	†	†

Bold values indicate the highest docking scores, representing the best binding energy for specific phytochemicals under various extraction methods.

†.: Not detected.

*.: Not applicable.

Epicatechin was better extracted in ultrasonic-assisted extraction processes, while it could not be extracted using supercritical carbon dioxide extraction (**Table 1**. No: 15). Similarly, caffeic acid and astragalus were only extracted using subcritical ethanol extraction (**Table 1**. No: 17 and No: 42). Daidzin, epicatechin gallate, piceid, isoquercitrin, nicotiflorin, and kaempferol were present in all extracts but could not be extracted in the supercritical carbon dioxide extraction processes (**Table 1**).

In summary, based on these results. the extraction method, solvent, duration, temperature, and pressure can be adjusted to obtain extracts enriched with different phytochemicals. This approach can lead to the creation of extracts with a rich content that may influence biological activity. Quinic acid is found in the highest amount in the UAE-E-2h extract, while it is present in the lowest amount in the Sc-400 atm extract. One of the most significant antioxidants, epicatechin gallate, is found in concentrations ranging from 0.826 to 1.193 across the extracts; interestingly, it is absent in extracts obtained by the SC-CO_2_ method. As for protocatechuic acid, the highest concentration is observed in the Sbc-EtOH-140-80 extract, while the lowest is found in extracts obtained via the SC-CO_2_ method. Catechin and chlorogenic acid have their highest content in extracts produced by the UAE method. Epicatechin, daidzin, isoquercitrin, nicotiflorin, and kaempferol are present in all extracts except those obtained using the supercritical CO_2_ method.

### 3.2 Antioxidant activity

There are various methods for determining antioxidant activities. Typically, the chemical complexity of extracts, which possess different functional groups, polarities, and chemical behaviors, produces varying results depending on the test used. Therefore, a multi-assay approach is considered more beneficial for evaluating the antioxidant potential of extracts. In this study, the DPPH radical scavenging activity, ABTS radical scavenging activity, and cupric ion reducing capacity (CUPRAC) were primarily employed. The total phenolic content, DPPH and ABTS radical scavenging activities, and Cu^2+^-Cu^+^ reducing capacity of the extracts were calculated, and the results are presented in **Table 2**.

**Table 2:** Radical Scavenging Activity (IC_50_ mg/mL) and Total Phenolic Content (mg GAE/g).

	DPPH (IC_50_ mg/mL)	ABTS (IC_50_ mg/mL)	CUPRAC (mg TE/mL)	Total Phenolic Content (mg GAE/g)
**BHA**	0.0021 ± 0.00015	0.0023 ± 0.00012		
**BHT**	0.0036 ± 0.00018	0.0032 ± 0.00015		
**Trolox**	0.0074 ± 0.0004	0.0043 ± 0.0002		
**UAE-M-2h**	0.0194± 0.00097^a^	0.0027 ± 0.00014	0.0138 ± 0.00069	199.75 ± 5
**UAE-E-2h**	0.0188± 0.00099^a^	0.0030 ± 0.00013	0.0125 ± 0.00061	192.42 ± 6
**UAE-M-4h**	0.0191± 0.001^a^	0.0030 ± 0.00014	0.0121 ± 0.00058^b,c^	213.44 ± 7^c^
**UAE-E-4h**	0.0185± 0.00098^a^	0.0049 ± 0.00018^a,b^	0.0072 ± 0.00039^b,c,d^	197.31 ± 5^d^
**Sbc-EtOH-140-60**	0.0182± 0.00096^a^	0.0050 ± 0.00014^a,b,c,d^	0.0075 ± 0.0004^c,d^	185.57 ± 4^d^
**Sbc-EtOH-140-80**	0.0173± 0.00087^a^	0.0049 ± 0.00015^a,b,d^	0.0084 ± 0.00046^c,d^	182.64 ± 5^b,d,e^
**Sc-90 atm**	0.0356 ± 0.00178^b,c,d,e,f,g^	0.0270 ± 0.00138	0.0088 ± 0.00045^c,d^	167.27 ± 5^b,c,d,e,f,g^
**Sc-400 atm**	0.0400 ± 0.002^b,c,d,e,f,g^	0.0275 ± 0.00143	0.0058 ± 0.00025^c,d,f,g,h^	137.09 ± 3^b,c,d,e,f,g,h^

The data represent the means ± SD of three independent experiments.

a: Statistically significant at p < 0.05 compared to BHA

b: Statistically significant at p < 0.05 compared to UAE-M-2h

c: Statistically significant at p < 0.05 compared to UAE-E-2h

d: Statistically significant at p < 0.05 compared to UAE-M-4h

e: Statistically significant at p < 0.05 compared to UAE-E-4h

f: Statistically significant at p < 0.05 compared to Sbc-EtOH-140-60

g: Statistically significant at p < 0.05 compared to Sbc-EtOH-140-80

h: Statistically significant at p < 0.05 compared to Sc-90 atm

Bold text highlights values that are statistically significantly different from others, or key results that hold critical importance in the context of the experiment.

For the DPPH radical scavenging activity, the IC_50_ values of the standard antioxidants BHA, BHT, and Trolox were found to be 0.0021, 0.0033, and 0.0074 mg/mL, respectively. The IC_50_ values for the DPPH radical scavenging activity of obtained extracts ranged between 0.0173 mg/mL and 0.0400 mg/mL. The highest DPPH free radical scavenging activities were observed in the extracts Sbc-EtOH-140-80 and Sbc-EtOH-140-60, with IC_50_ values of 0.0173 mg/mL and 0.0182 mg/mL, respectively. Antioxidant activity increased with increasing extract concentration.

For the ABTS radical scavenging activity, the IC_50_ values of the standard antioxidants BHA, BHT, and Trolox were found to be 0.0023, 0.0032, and 0.0043 mg/mL, respectively. The IC_50_ values for the ABTS radical scavenging activity of Tartarium root plant extracts ranged from 0.0027 mg/mL to 0.0275 mg/mL. The Tartarium root plant extracts exhibited higher activity than BHT and Trolox and showed activity closer to that of BHA. Antioxidant activity also increased with the concentration of the extracts.

For the Cu^2+^-Cu^+^ reducing activity, the Trolox equivalents of the plant extracts ranged between 0.0058 and 0.0138 mg TE/mL. The highest values were found in the extracts UAE-E-2h, UAE-M-2h, and UAE-M-4h, with values of 0.0138, 0.0125, and 0.0121 mg TE/mL, respectively. The total phenolic content in the plant extracts was calculated as gallic acid equivalents. The lowest total phenolic content was found in the Sbc-EtOH-140-80 plant extract (182.64 mg GAE/mL), while the highest was found in the UAE-M-4h extract (213.44 mg GAE/mL). According to the antioxidant data obtained, the UAE method showed superiority over other methods.

### 3.3 *In vitro* antimicrobial activity

The MIC and MBC values of test microorganisms against different extracts of *Rheum tataricum* L. are showed in **Table 3**. The MIC values of the different extracts ranged from 31.25 μg/mL to 250 μg/mL. *E. faecalis* was one of the most sensitive bacteria to the extracts, with a MIC of 31.25 μg/mL. For the UAE-MeOH-2h extract, the MIC values were 125 μg/mL for *E. faecalis* and 250 μg/mL for *S. aureus*, *E. coli*, and *P. aeruginosa*. In the UAE-MeOH-4h extract, the MIC values were 125 μg/mL for *S. aureus*, *E. faecalis*, and *E. coli*, and 250 μg/mL for *P. aeruginosa*. In the UAE-EtOH-2h extract, the MIC values were 125 μg/mL for E. faecalis and P. aeruginosa, and 250 μg/mL for *S. aureus* and *E. coli*. Conversely, for the UAE-EtOH-4h extract, the MIC values were 125 μg/mL for *S. aureus*, *E. faecalis*, and *P. aeruginosa*, and 250 μg/mL for *E. coli*. The sbcEtOH-E 140-60 and 140-80 extracts exhibited similar antimicrobial activity against the tested microorganisms. The MIC values for these extracts were 31.25 μg/mL for *E. faecalis*, 125 μg/mL for *S. aureus* and P*. aeruginosa*, and 250 μg/mL for *E. coli*. The Sc-90 atm extract exhibited greater antibacterial activity than the Sc-400 atm extract. The MBC values for the different extracts of *Rheum tataricum* L. ranged from 31.5 μg/mL to 1000 μg/mL against the tested bacteria. Based on the MIC and MBC results, it can be concluded that these extracts can be considered potent antimicrobials, particularly against *E. faecalis*.

**Table 3:** Presents the MIC (μg/mL) and MBC (μg/mL) values of the extracts against Gram-positive and Gram-negative pathogenic bacteria.

	*S. aureus*		*E. faecalis*		*P. aeruginosa*		*E. coli*	
	MIC	MBC	MIC	MBC	MIC	MBC	MIC	MBC
**UAE-EtOH-2h**	250	250	125	125	125	1000	250	500
**UAE-EtOH-4h**	125	250	125	500	125	500	250	500
**UAE-MeOH-2h**	250	500	125	500	250	500	250	500
**UAE-MeOH-4h**	125	250	125	500	125	1000	250	500
**sbcEtOH-E** **140-60**	125	500	31.25	31.25	125	1000	250	500
**sbcEtOH-E** **140-80**	125	250	31.25	31.25	125	500	250	500
**Sc-90 atm**	250	500	250	500	1000	250	250	500
**Sc-400 atm**	125	500	31.25	125	125	500	125	125
**Ampicillin**	*		*		31.25		3.9	

Bold text emphasizes the minimum inhibitory concentrations (MIC) or minimum bactericidal concentrations (MBC) that demonstrate the highest antibacterial activity among the tested extracts.

The symbol "*" indicates that the extract was effective at all tested concentrations against the specific bacteria.

In this study, different extracts of *Rheum tataricum* L. were investigated for their antibacterial effects on some Gram-positive and Gram-negative bacteria. According to the results, it was determined that the extracts prepared with UAE-MeOH-2h-4h, UAE-EtOH-2h-4h, Sbc-EtOH-E-140-60-80, Sc-90 atm, and Sc-400 atm showed antibacterial activity against both Gram-positive and Gram-negative bacteria at varying rates (MIC range: 31.25 to 250 μg/mL). Among the Gram-negative bacteria tested, the green method (Sc-400) exhibited the strongest antibacterial effect on *E. coli* compared to the other extracts. The Sc-90 atm and Sc-400 atm extracts showed varying antibacterial activities due to changes in the pressure parameters during extraction, which affected the extract content. Except for UAE-MeOH-2h and Sc-90 atm (MIC value of 250 μg/mL), the other extracts (MIC value of 125 μg/mL) showed similar antibacterial effects against *P. aeruginosa*.

For the Gram-positive bacteria, the Sbc-EtOH-E 140-60, 140-80, and Sc-400 atm extracts (MIC value of 31.25 μg/mL) exhibited strong antibacterial effects on *E. faecalis* compared to the other extracts. The best antibacterial effect against *S. aureus* was observed in extracts other than UAE-MeOH-2h and UAE-EtOH-2h. The antibacterial activity of plant essential oils or extracts is generally stronger against Gram-positive bacteria than Gram-negative bacteria, which is attributed to the more complex cell wall structure of Gram-negative bacteria (Puupponen-Pimiä et al., 2001; Nazzaro et al., 2013).

The sbcEtOH-E 140-60 and sbcEtOH-E 140-80 extracts showed a notably strong antibacterial effect against *E. faecalis*. This effect, compared to other extracts, is attributed to their higher content of compounds such as protocatechuic acid, protocatechuic aldehyde, 4-OH benzoic acid, p-coumaric acid, hesperidin, rutin, and gallic acid, which are more abundant in these extracts than in those obtained by other extraction methods.

In a study by Onem et al (Önem et al., 2020), the methanolic, methanol-chloroform, and aqueous extracts of *Rheum ribes* L. were tested against S. aureus, methicillin-resistant *S. aureus* (MRSA), *Bacillus cereus*, *Enterococcus faecalis*, and *Listeria monocytogenes*. They found that the methanolic extract exhibited the most antibacterial activity against MRSA and B. cereus. Similarly, Alan et al. tested chloroform, hexane, acetone, ethanol, and methanol extracts from different parts of *Rheum ribes* L. against various microorganisms, with the most significant activity observed against *Bacillus subtilis* ATCC 6633 and *Enterobacter aerogenes* ATCC 13048 in ethanol and methanol extracts obtained from the root, stalk, and seed (Alan et al., 2013). Rolta et al. evaluated the antimicrobial effects of methanolic extracts and different solvent fractions (n-hexane, chloroform, ethyl acetate, and residual aqueous) of *Rheum emodi* against *E. coli*, *S. aureus*, and *K. pneumoniae*. The MIC values of the chloroform sub-fraction were 1.95, 3.91, and 15.62 μg/mL against *S. aureus*, *K. pneumoniae*, and *E. coli*, respectively (Rolta et al., 2020). Additionally, another study found that ethanol and water extracts of *Rheum ribes* L. roots had antibacterial effects against *S. aureus* (Alaadin et al., 2007). Chen et al. demonstrated the antibacterial activity of different extracts of *Rheum palmatum* L. (Turkey rhubarb) roots. The liquid dilution MIC values of methanolic, aqueous, and ethyl acetate extracts inhibited the growth of A. baylyi at concentrations of 459, 1879, and 686 μg/mL, respectively, and *P. aeruginosa* at 230, 939, and 194 μg/mL, respectively. The chloroform and hexane extracts of *Rheum palmatum* L. also showed good antibacterial activity against *P. aeruginosa* (MIC values of 128 and 96 μg/mL, respectively) (Chen and Cock, 2022). The prepared extracts are rich in compounds such as catechin, chlorogenic acid, epicatechin, cyranoside, and gallic acid. According to the literature, these compounds are known to exhibit potent activity against *S. aureus*, *E. coli*, *P. aeruginosa*, and *E. faecalis* bacteria.

### 3.4 Molecular modelling

Molecular modeling studies provide significant insights into the interaction between compounds present in the prepared extracts and disease-associated proteins. In this study, interactions between the most abundant compounds in the plant extracts and proteins found in *S. aureus*, *E. coli*, *P. aeruginosa*, and *E. faecalis*—specifically, proteins 1JIJ, 4WUB, 2UV0, and 6QXS, respectively—were investigated.

**Figure 2:**
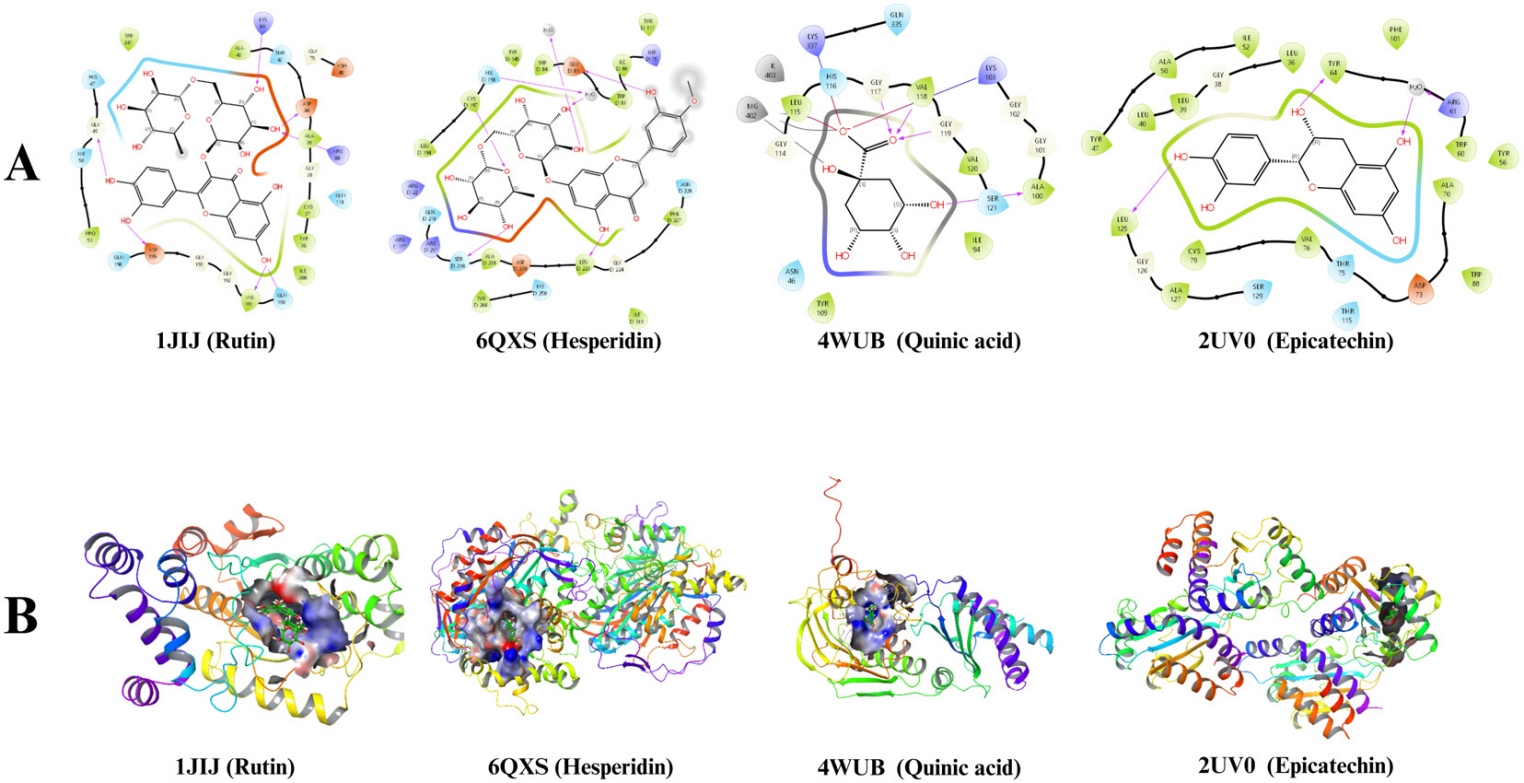
2D **(A)** and 3D **(B)** binding interactions of compounds in the active site of proteins.

Upon analyzing the molecular docking scores, it was found that rutin showed a docking score of -10.107 for the 1JIJ protein in *S. aureus*, hesperidin scored -8.746 for the 6QXS protein in *E. faecalis*, epicatechin gallate scored -10.031 for the 4WUB protein in *E. coli*, and epicatechin scored -8.176 for the 2UV0 protein in *P. aeruginosa* (**Table 4**). **Figure 2** illustrates the 2D and 3D interaction images of the molecules with the highest molecular docking scores with their respective proteins.

**Table 4:** Molecular docking scores and binding modes of compounds with the 1JIJ, 4WUB, 2UV0, and 6QXS receptor.

Compound	1JIJ (*S. aureus*)	6QXS (*E. faecalis*)	4WUB (*E. coli*)	2UV0(*P. aeruginosa*)
Cyranoside	-6.902	-7.052	-6.692	-6.904
Catechin	-8.330	-7.071	-7.638	**-8.176**
Quinic acid	-7.177	-7.068	**-10.500**	-6.455
Gallic acid	-7.069	-6.165	-9.856	-5.816
Protocatechuic acid	-6.890	-6.231	-9.697	-6.805
Chlorogenic acid	-8.241	-7.098	-10.691	-7.501
4-OH Benzoic acid	-6.495	-6.864	-9.934	-6.695
Epicatechin	-8.330	-7.071	-7.638	**-8.176**
Epicatechin gallate	-8.875	-7.546	**-10.031**	-6.124
Rutin	**-10.107**	-7.983	-8.779	-6.502
Hesperidin	-8.474	**-8.746**	-7.425	-5.932

Bold values indicate key statistical results, such as optimal extraction yields or the most significant biochemical activities among the methods tested.

## 4 Conclusion

In this study, extracts of *Rheum tataricum* L. were obtained using supercritical CO_2_, subcritical ethanol, and ultrasound assisted extraction methods under varying temperatures and solvents. According to the LC-MS/MS data, catechin, epicatechin, cyranoside, and chlorogenic acid were identified as the predominant compounds among the 53 phytochemicals detected in the extracts. All extracts exhibited antibacterial activity against four different bacteria. Our results indicate the high antimicrobial potential of *Rheum tataricum* L. extracts, particularly against *E. faecalis* as a Gram-positive bacterium (with a MIC value of 31.25 μg/mL) when using the Sc-400 atm and Sbc-EtOH-E 140-60, 140-80 extraction methods. Upon analyzing all the data, the ultrasound assisted extraction method emerged as cheap and easy-to-apply technique, outperforming the other methods. Additionally, different methods may be preferred depending on the target compound to be isolated. Based on the obtained data, *Rheum tataricum L.* roots extract demonstrate a wide range of application potential, extending from the food industry to pharmaceutical applications. Among the extraction methods used, UAE stands out as a preferred technique due to its ease of application and cost-effectiveness. Additionally, the choice of extraction method can be tailored depending on the desired active compounds. Furthermore, given the strong antibacterial properties exhibited by the extracts, they may be considered as natural antimicrobial agents in pharmaceutical products.

## Data Availability

The datasets presented in this study can be found in online repositories. The names of the repository/repositories and accession number(s) can be found in the article/**Supplementary Material**.
