# Supplementary material for: Simultaneous quantitative screening of 53 phytochemicals from Rheum tataricum L. roots: a comparative study of supercritical CO2, subcritical ethanol, and ultrasound-assisted extraction for enhanced antioxidant, antibacterial activities, and molecular docking study
Source: Front Plant Sci. 2024 Dec 6;15:1513875. doi: 10.3389/fpls.2024.1513875 (PMC11662978; doi:10.3389/fpls.2024.1513875)
Supplement: Supplementary file 1 [file DataSheet1.docx]

***SUPPLEMENTARY MATERIAL***

***FOR***

**Simultaneous Quantitative Screening of 53 Phytochemicals from *Rheum tataricum* L. Roots: A Comparative Study of Supercritical CO_2_, Subcritical Ethanol, and Ultrasound Assisted Extraction for Enhanced Antioxidant and Antibacterial Activities**

Madina Amangeldinova^1,2^, Mehmet Ersatır^3^, Adem Necip^4^, Mustafa Abdullah Yilmaz^5,6^, Mehmet Cimentepe^7^, Nataliya Kudrina^1,2^ , Nina V. Terletskaya^1,2^ and Metin Yildirim^8^,*

1. Faculty of Biology and Biotechnology, al-Farabi Kazakh National University, Al-Farabi Avenue 71, Almaty 050040, Kazakhstan;

2. Institute of Genetics and Physiology, Al-Farabi 93, Almaty 050040, Kazakhstan

3. Department of Chemistry, Faculty of Art and Science, Cukurova University, Adana, Türkiye

4. Department of Pharmacy Services, Vocational School of Health Services, Harran University, Sanliurfa, Türkiye

5. Dicle University Science and Technology Research and Application Center, 21280, Diyarbakir, Türkiye

6. Dicle University, Faculty of Pharmacy, Department of Analytical Chemistry, 21280, Diyarbakir, Türkiye

7. Department of Pharmaceutical Microbiology, Faculty of Pharmacy, Harran University, Sanliurfa, Tü-rkiye

8. Department of Biochemistry, Faculty of Pharmacy, Harran University, Sanliurfa, Türkiye

*Correspondence: metinyildirim4@gmail.com; Tel.: +905375274443 (M.Y.)

| ***Supplementary Data*** |  | **Page Number** |
| --- | --- | --- |
| **Fig. S1.** TIC (Total Ion Chromatogram) chromatogram of the extract obtained from the ultrasound-assisted extraction of *Rheum tataricum* L. roots in ethanol for 2 h |  | S3 |
| **Fig. S2.** TIC (Total Ion Chromatogram) chromatogram of the extract obtained from the ultrasound-assisted extraction of *Rheum tataricum* L. roots in ethanol for 4h |  | S4 |
| **Fig. S3.** TIC (Total Ion Chromatogram) chromatogram of the extract obtained from the ultrasound-assisted extraction of *Rheum tataricum* L. roots in methanol for 2h |  | S5 |
| **Fig. S4.** TIC (Total Ion Chromatogram) chromatogram of the extract obtained from the ultrasound-assisted extraction of *Rheum tataricum* L. roots in methanol for 4h |  | S6 |
| **Fig. S5.** TIC (Total Ion Chromatogram) chromatogram of the extract obtained from Rheum tataricum L. roots by supercritical CO_2_ extraction at 90 atm pressure and 60^o^C temperature |  | S7 |
| **Fig. S6.** TIC (Total Ion Chromatogram) chromatogram of the extract obtained from Rheum tataricum L. roots by supercritical CO_2_ extraction at 200 atm pressure and 60^o^C temperature |  | S8 |
| **Fig. S7.** TIC (Total Ion Chromatogram) chromatogram of the extract obtained from *Rheum tataricum* L. roots by subcritical ethanol extraction at 140 atm pressure and 60^o^C temperature |  | S9 |
| **Fig. S8.** TIC (Total Ion Chromatogram) chromatogram of the extract obtained from *Rheum tataricum* L. roots by subcritical ethanol extraction at 140 atm pressure and 80^o^C temperature |  | S10 |
| **Table 1.** Analytical method validation parameters that belong to the LC-MS/MS method  Mass spectrometer and chromatograph conditions for LC-MS/MS analysis  Fig S9. TIC (Total Ion Chromatogram) chromatogram of standard phenolic compounds |  | S11  S14 |

**
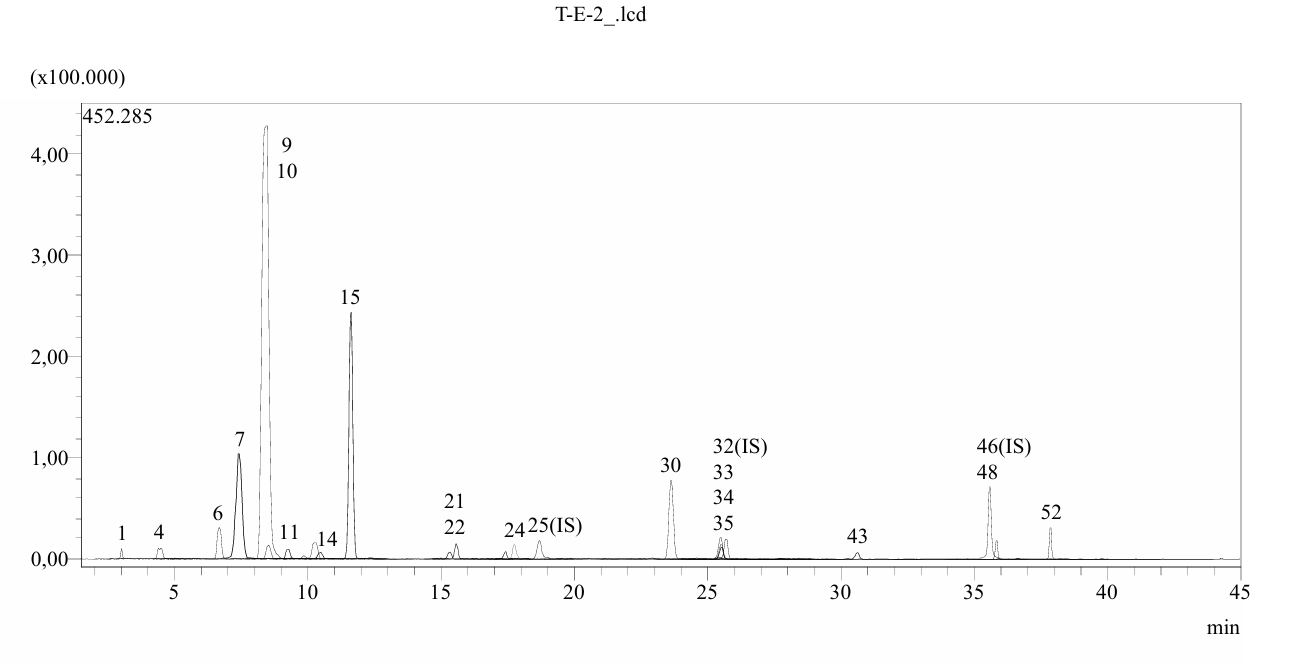
**

**Fig. S1.** TIC (Total Ion Chromatogram) chromatogram of the extract obtained from the ultrasound-assisted extraction of *Rheum tataricum* L. roots in ethanol for 2h

1:Quinic acid, 4: Gallic acid, 6: Protocatechuic acid, 7: Catechin, 9: Chlorogenic acid, 10: Protocatechuic aldehyde, 11: Tannic acid, 14: 4-OH Benzoic acid, 15: Epicatechin, 21: Daidzin, 22: Epicatechin gallate, 24: p-Coumaric acid, 25: Ferulic acid D3, 30: Cynaroside, 32: Rutin, 33: Rutin D3, 34: isoquercitrin, 35: Hesperidin, 43: Nicotiflorin, 46: Quercetin D3, 48: Naringenin, 52: Kaempferol.

**
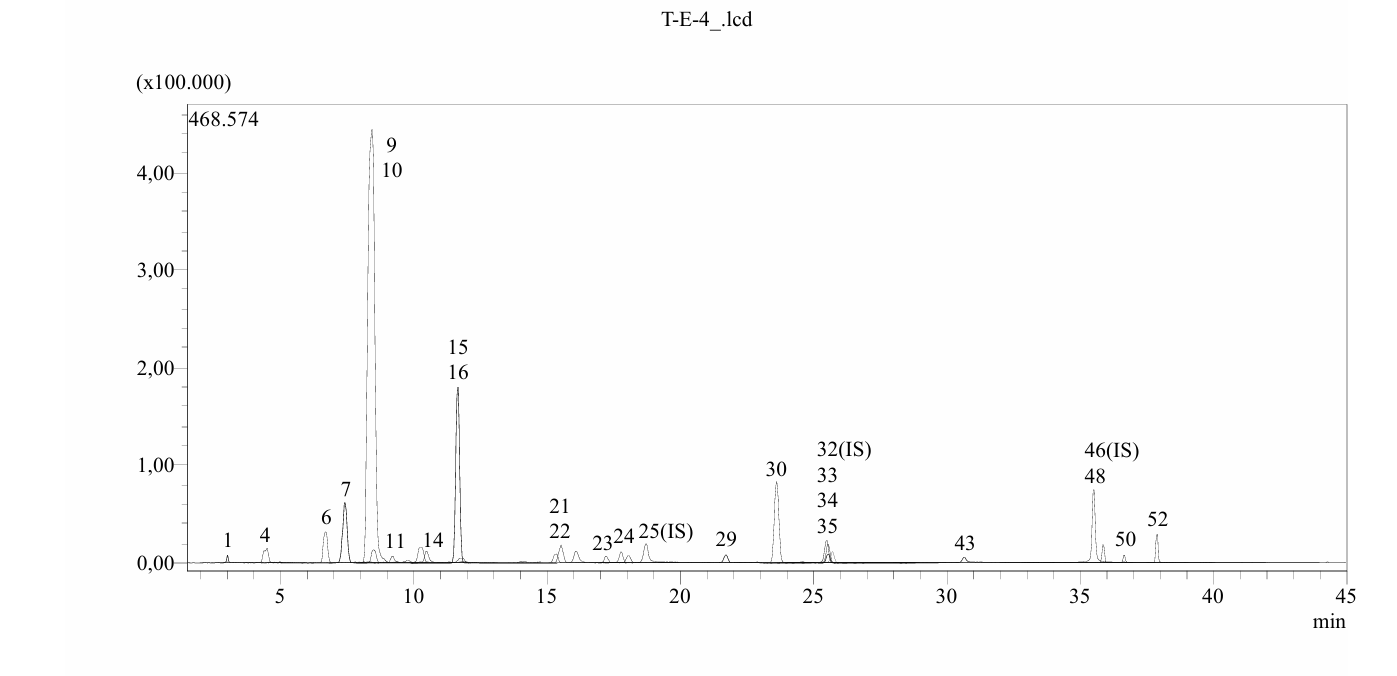
**

**Fig. S2.** TIC (Total Ion Chromatogram) chromatogram of the extract obtained from the ultrasound-assisted extraction of *Rheum tataricum* L. roots in ethanol for 4h

1:Quinic acid, 4: Gallic acid, 6: Protocatechuic acid, 7: Catechin, 9: Chlorogenic acid, 10: Protocatechuic aldehyde, 11: Tannic acid, 14: 4-OH Benzoic acid, 15: Epicatechin, 16: Vanilic acid, 21: Daidzin, 22: Epicatechin gallate, 23: Piceid, 24: p-Coumaric acid, 25: Ferulic acid D3, 29: Salicylic acid, 30: Cynaroside, 32: Rutin, 33: Rutin D3, 34: isoquercitrin, 35: Hesperidin, 43: Nicotiflorin, 46: Quercetin D3, 48: Naringenin, 50: Luteolin, 52: Kaempferol

**
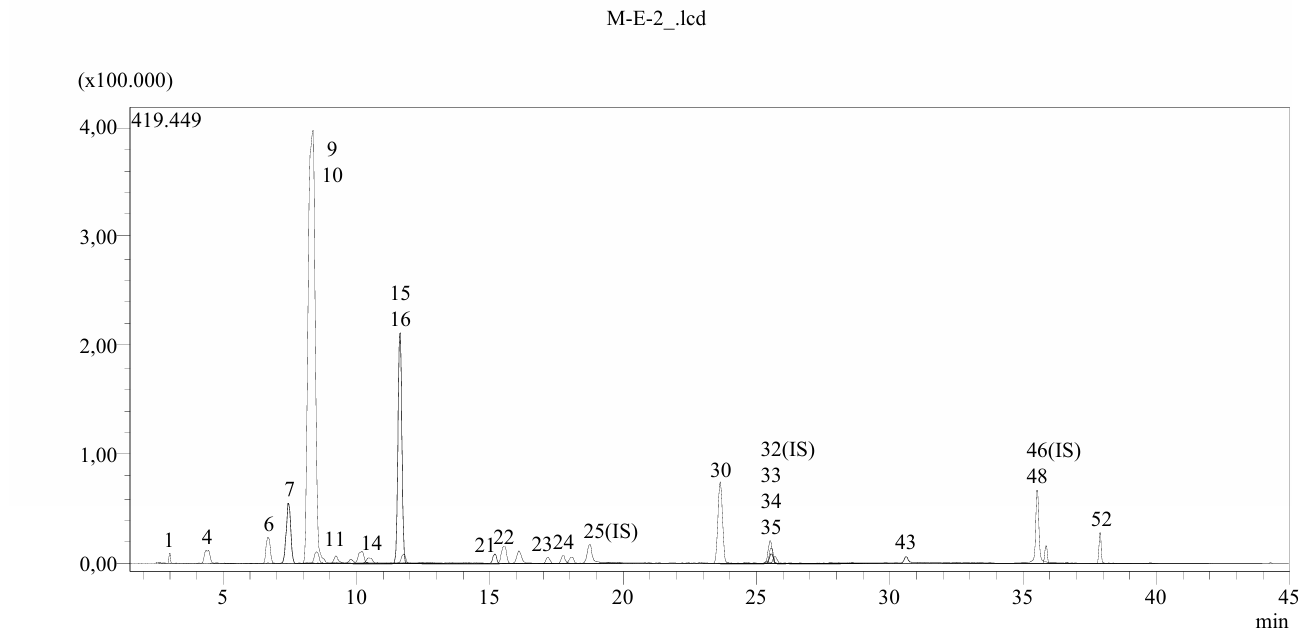
**

**Fig. S3.** TIC (Total Ion Chromatogram) chromatogram of the extract obtained from the ultrasound-assisted extraction of *Rheum tataricum* L. roots in methanol for 2h

1:Quinic acid, 4: Gallic acid, 6: Protocatechuic acid, 7: Catechin, 9: Chlorogenic acid, 10: Protocatechuic aldehyde, 11: Tannic acid, 14: 4-OH Benzoic acid, 15: Epicatechin, 21: Daidzin, 22: Epicatechin gallate, 23: Piceid, 24: p-Coumaric acid, 25: Ferulic acid D3, 30: Cynaroside, 32: Rutin, 33: Rutin D3, 34: isoquercitrin, 35: Hesperidin, 43: Nicotiflorin, 46: Quercetin D3, 48: Naringenin, 52: Kaempferol

**
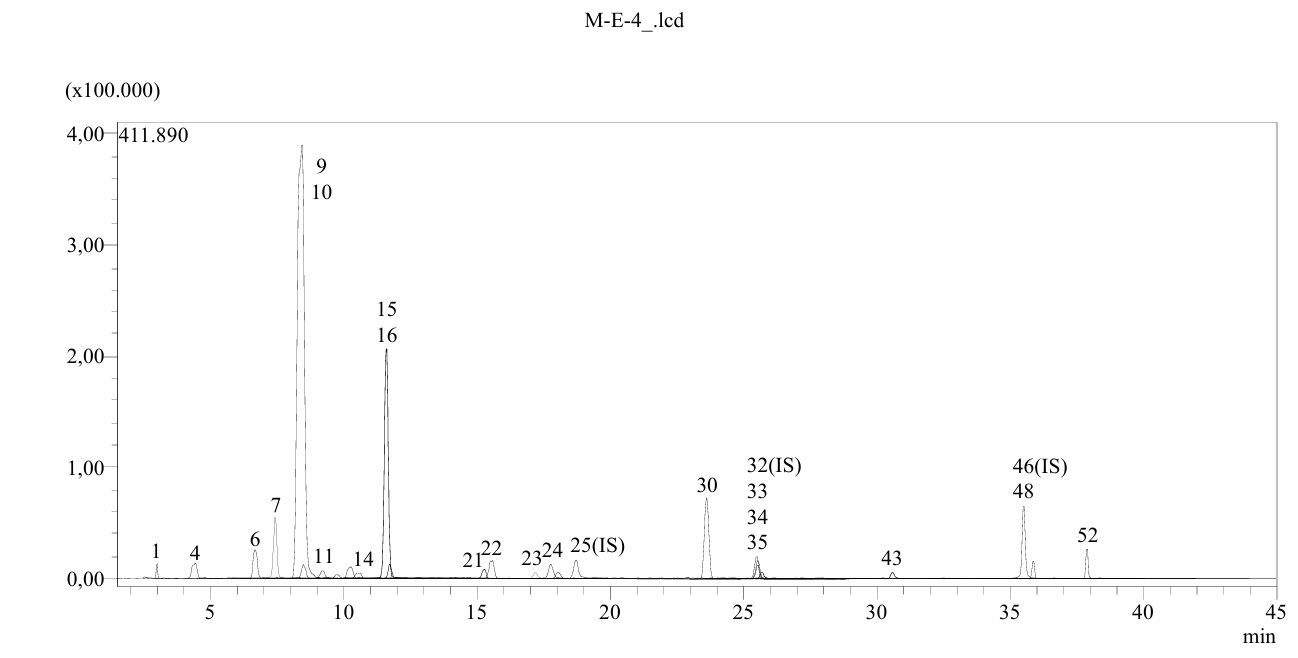
**

**Fig. S4.** TIC (Total Ion Chromatogram) chromatogram of the extract obtained from the ultrasound-assisted extraction of *Rheum tataricum* L. roots in methanol for 4h

1:Quinic acid, 4: Gallic acid, 6: Protocatechuic acid, 7: Catechin, 9: Chlorogenic acid, 10: Protocatechuic aldehyde, 11: Tannic acid, 14: 4-OH Benzoic acid, 15: Epicatechin, 16: Vanilic acid, 21: Daidzin, 22: Epicatechin gallate, 23: Piceid, 24: p-Coumaric acid, 25: Ferulic acid D3, 30: Cynaroside, 32: Rutin, 33: Rutin D3, 34: isoquercitrin, 35: Hesperidin, 43: Nicotiflorin, 46: Quercetin D3, 48: Naringenin, 52: Kaempferol

**
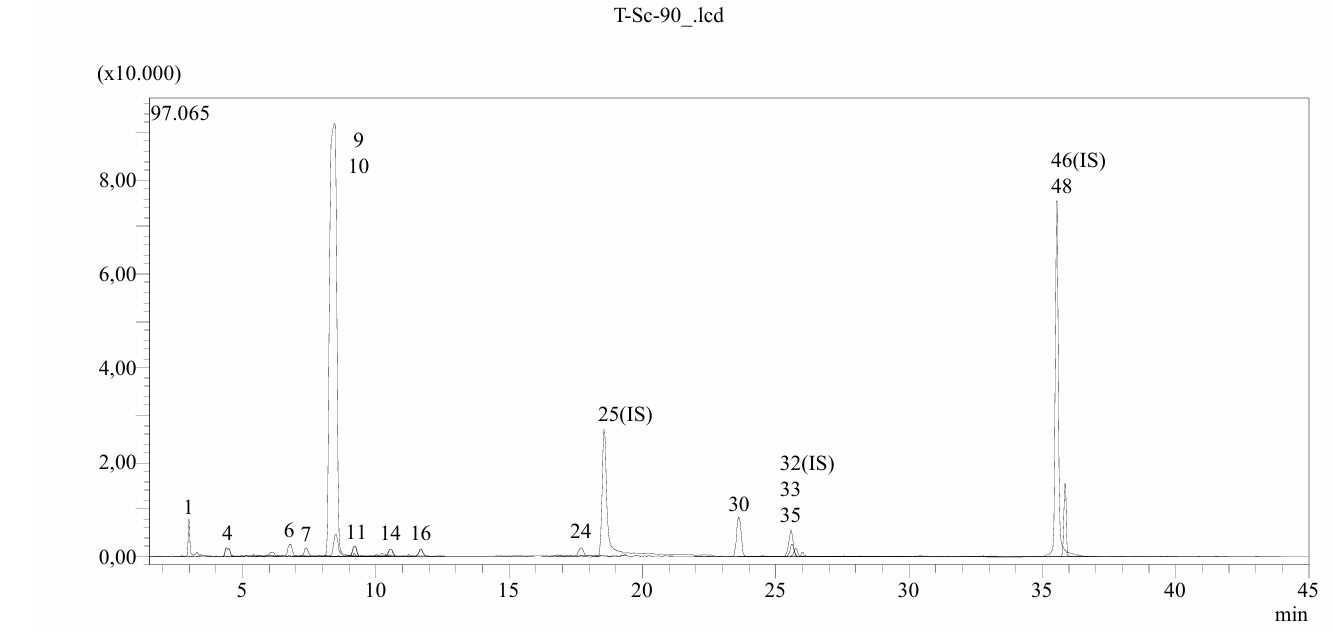
**

**Fig. S5.** TIC (Total Ion Chromatogram) chromatogram of the extract obtained from Rheum tataricum L. roots by supercritical CO_2_ extraction at 90 atm pressure and 60^o^C temperature

1:Quinic acid, 4: Gallic acid, 6: Protocatechuic acid, 7: Catechin, 9: Chlorogenic acid, 10: Protocatechuic aldehyde, 11: Tannic acid, 14: 4-OH Benzoic acid, 16: Vanilic acid, 24: p-Coumaric acid, 25: Ferulic acid D3, 30: Cynaroside, 32: Rutin, 33: Rutin D3, 35: Hesperidin, 46: Quercetin D3, 48: Naringenin

**
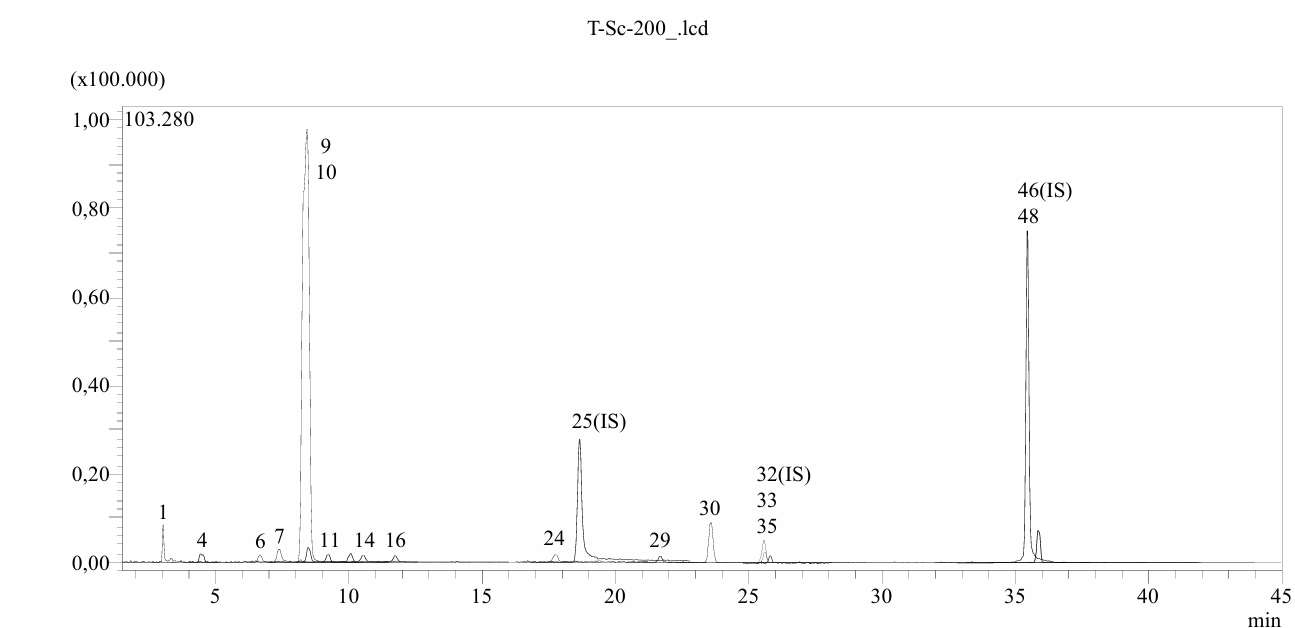
**

**Fig. S6.** TIC (Total Ion Chromatogram) chromatogram of the extract obtained from Rheum tataricum L. roots by supercritical CO_2_ extraction at 400 atm pressure and 60^o^C temperature

1:Quinic acid, 4: Gallic acid, 6: Protocatechuic acid, 7: Catechin, 9: Chlorogenic acid, 10: Protocatechuic aldehyde, 11: Tannic acid, 14: 4-OH Benzoic acid, 16: Vanilic acid, 24: p-Coumaric acid, 25: Ferulic acid D3, 29: Salicylic acid, 30: Cynaroside, 32: Rutin, 33: Rutin D3, 35: Hesperidin, 46: Quercetin D3, 48: Naringenin

**
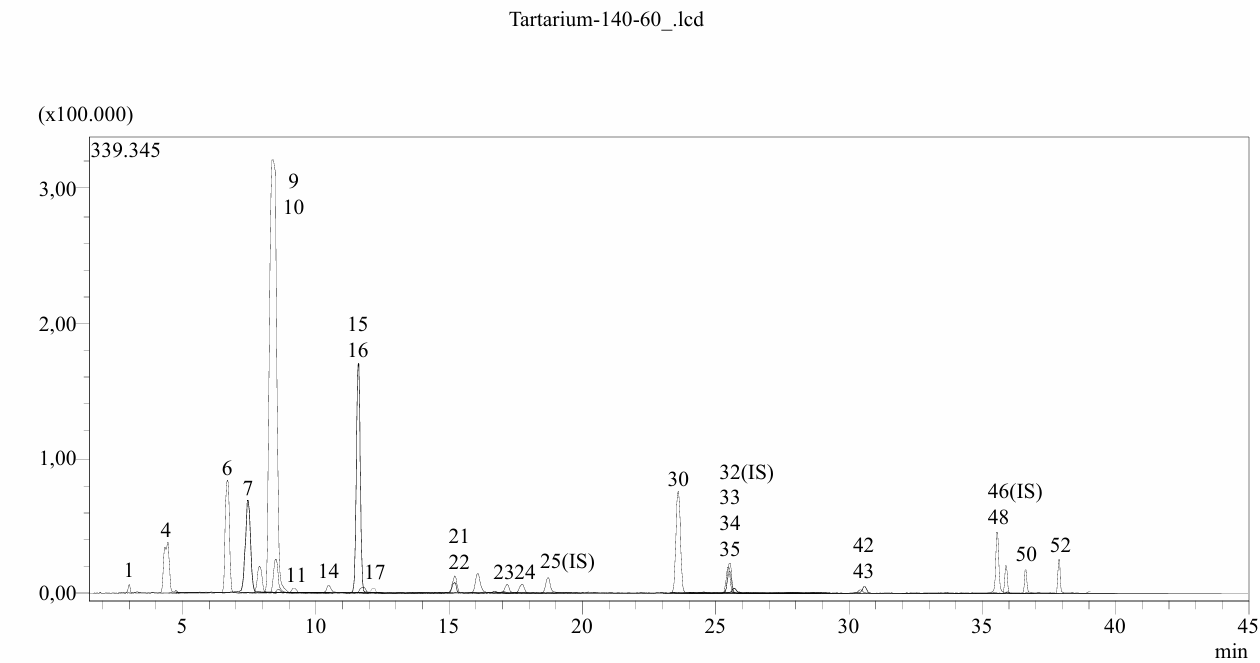
**

**Fig. S7.** TIC (Total Ion Chromatogram) chromatogram of the extract obtained from *Rheum tataricum* L. roots by subcritical ethanol extraction at 140 atm pressure and 60^o^C temperature

1:Quinic acid, 4: Gallic acid, 6: Protocatechuic acid, 7: Catechin, 9: Chlorogenic acid, 10: Protocatechuic aldehyde, 11: Tannic acid, 14: 4-OH Benzoic acid, 15: Epicatechin, 16: Vanilic acid, 17: Caffeic acid, 21: Daidzin, 22: Epicatechin gallate, 23: Piceid, 24: p-Coumaric acid, 25: Ferulic acid D3, 30: Cynaroside, 32: Rutin, 33: Rutin D3, 34: isoquercitrin, 35: Hesperidin, 42: Astragalin, 43: Nicotiflorin, 46: Quercetin D3, 48: Naringenin, 50: Luteolin, 52: Kaempferol

**
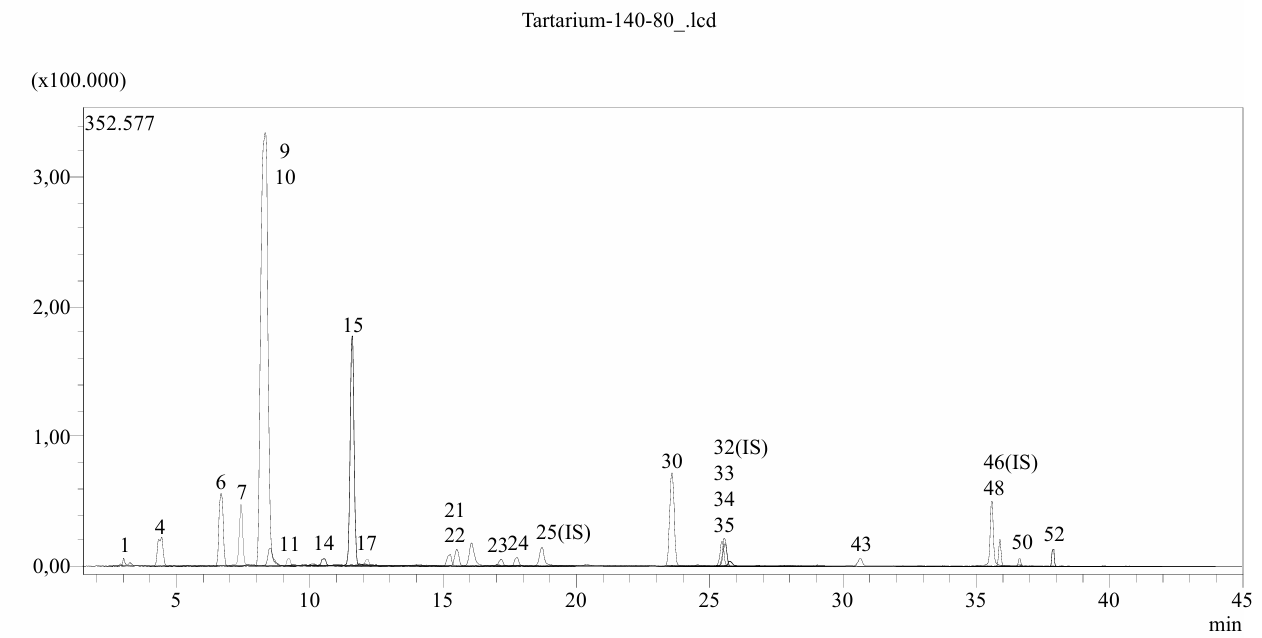
**

**Fig. S8.** TIC (Total Ion Chromatogram) chromatogram of the extract obtained from *Rheum tataricum* L. roots by subcritical ethanol extraction at 140 atm pressure and 80^o^C temperature

1:Quinic acid, 4: Gallic acid, 6: Protocatechuic acid, 7: Catechin, 9: Chlorogenic acid, 10: Protocatechuic aldehyde, 11: Tannic acid, 14: 4-OH Benzoic acid, 15: Epicatechin, 17: Caffeic acid, 21: Daidzin, 22: Epicatechin gallate, 23: Piceid, 24: p-Coumaric acid, 25: Ferulic acid D3, 30: Cynaroside, 32: Rutin, 33: Rutin D3, 34: isoquercitrin, 35: Hesperidin, 43: Nicotiflorin, 46: Quercetin D3, 48: Naringenin, 50: Luteolin, 52: Kaempferol

**Table 1.** Analytical method validation parameters that belong to the LC-MS/MS method

| No | Analytes | RT*^a^* | M.I. (m/z)*^b^* | F.I. (m/z)*^c^* | Ion. mode | Equation | *r^2d^* | *RSD*%*^e^* | | Linearity Range (mg/L) | *LOD*/*LOQ* (µg/L)*^f^* | Recovery (%) | | *U^g^* | Gr. No*^i^* |
| --- | --- | --- | --- | --- | --- | --- | --- | --- | --- | --- | --- | --- | --- | --- | --- |
|  |  |  |  |  |  |  |  | Interday | Intraday |  |  | Interday | Intraday |  |  |
| 1 | Quinic acid | 3.0 | 190.8 | 93.0 | Neg | *y*=-0.0129989+2.97989*×* | 0.996 | 0.69 | 0.51 | 0.1-5 | 25.7/33.3 | 1.0011 | 1.0083 | 0.0372 | 1 |
| 2 | Fumaric aid | 3.9 | 115.2 | 40.9 | Neg | *y*=-0.0817862+1.03467*×* | 0.995 | 1.05 | 1.02 | 1-50 | 135.7/167.9 | 0.9963 | 1.0016 | 0.0091 | 1 |
| 3 | Aconitic acid | 4.0 | 172.8 | 129.0 | Neg | *y*=-0.7014530+32.9994*×* | 0.971 | 2.07 | 0.93 | 0.1-5 | 16.4/31.4 | 0.9968 | 1.0068 | 0.0247 | 1 |
| 4 | Gallic acid | 4.4 | 168.8 | 79.0 | Neg | *y*=0.0547697+20.8152*×* | 0.999 | 1.60 | 0.81 | 0.1-5 | 13.2/17.0 | 1.0010 | 0.9947 | 0.0112 | 1 |
| 5 | Epigallocatechin | 6.7 | 304.8 | 219.0 | Neg | *y*=-0.00494986+0.0483704*×* | 0.998 | 1.22 | 0.73 | 1-50 | 237.5/265.9 | 0.9969 | 1.0040 | 0.0184 | 3 |
| 6 | Protocatechuic acid | 6.8 | 152.8 | 108.0 | Neg | *y*=0.211373+12.8622*×* | 0.957 | 1.43 | 0.76 | 0.1-5 | 21.9/38.6 | 0.9972 | 1.0055 | 0.0350 | 1 |
| 7 | Catechin | 7.4 | 288.8 | 203.1 | Neg | *y*=-0.00370053+0.431369*×* | 0.999 | 2.14 | 1.08 | 0.2-10 | 55.0/78.0 | 1.0024 | 1.0045 | 0.0221 | 3 |
| 8 | Gentisic acid | 8.3 | 152.8 | 109.0 | Neg | *y*=-0.0238983+12.1494*×* | 0.997 | 1.81 | 1.22 | 0.1-5 | 18.5/28.2 | 0.9963 | 1.0077 | 0.0167 | 1 |
| 9 | Chlorogenic acid | 8.4 | 353.0 | 85.0 | Neg | *y*=0.289983+36.3926*×* | 0.995 | 2.15 | 1.52 | 0.1-5 | 13.1/17.6 | 1.0000 | 1.0023 | 0.0213 | 1 |
| 10 | Protocatechuic aldehyde | 8.5 | 137.2 | 92.0 | Neg | *y*=0.257085+25.4657*×* | 0.996 | 2.08 | 0.57 | 0.1-5 | 15.4/22.2 | 1.0002 | 0.9988 | 0.0396 | 1 |
| 11 | Tannic acid | 9.2 | 182.8 | 78.0 | Neg | *y*=0.0126307+26.9263*×* | 0.999 | 2.40 | 1.16 | 0.05-2.5 | 15.3/22.7 | 0.9970 | 0.9950 | 0.0190 | 1 |
| 12 | Epigallocatechin gallate | 9.4 | 457.0 | 305.1 | Neg | *y*=-0.0380744+1.61233*×* | 0.999 | 1.30 | 0.63 | 0.2-10 | 61.0/86.0 | 0.9981 | 1.0079 | 0.0147 | 3 |
| 13 | 1,5-dicaffeoylquinic acid | 9.8 | 515.0 | 191.0 | Neg | *y*=-0.0164044+16.6535*×* | 0.999 | 2.42 | 1.48 | 0.1-5 | 5.8/9.4 | 0.9983 | 0.9997 | 0.0306 | 1 |
| 14 | 4-OH Benzoic acid | 10.5 | 137,2 | 65.0 | Neg | *y*=-0.0240747+5.06492*×* | 0.999 | 1.24 | 0.97 | 0.2-10 | 68.4/88.1 | 1.0032 | 1.0068 | 0.0237 | 1 |
| 15 | Epicatechin | 11.6 | 289.0 | 203.0 | Neg | *y*=-0.0172078+0.0833424*×* | 0.996 | 1.47 | 0.62 | 1-50 | 139.6/161.6 | 1.0013 | 1.0012 | 0.0221 | 3 |
| 16 | Vanilic acid | 11.8 | 166.8 | 108.0 | Neg | *y*=-0.0480183+0.779564*×* | 0.999 | 1.92 | 0.76 | 1-50 | 141.9/164.9 | 1.0022 | 0.9998 | 0.0145 | 1 |
| 17 | Caffeic acid | 12.1 | 179.0 | 134.0 | Neg | *y*=0.120319+95.4610*×* | 0.999 | 1.11 | 1.25 | 0.05-2.5 | 7.7/9.5 | 1.0015 | 1.0042 | 0.0152 | 1 |
| 18 | Syringic acid | 12.6 | 196.8 | 166.9 | Neg | *y*=-0.0458599+0.663948*×* | 0.998 | 1.18 | 1.09 | 1-50 | 82.3/104.5 | 1.0006 | 1.0072 | 0.0129 | 1 |
| 19 | Vanillin | 13.9 | 153.1 | 125.0 | Poz | *y*=0.00185898+20.7382*×* | 0.996 | 1.10 | 0.85 | 0.1-5 | 24.5/30.4 | 1.0009 | 0.9967 | 0.0122 | 1 |
| 20 | Syringic aldehyde | 14.6 | 181.0 | 151.1 | Neg | *y*=-0.0128684+7.90153*×* | 0.999 | 2.51 | 0.77 | 0.4-20 | 19.7/28.0 | 1.0001 | 0.9964 | 0.0215 | 1 |
| 21 | Daidzin | 15.2 | 417.1 | 199.0 | Poz | *y*=9.45747+152.338*×* | 0.996 | 2.25 | 1.32 | 0.05-2.5 | 7.0/9.5 | 0.9955 | 1.0017 | 0.0202 | 2 |
| 22 | Epicatechin gallate | 15.5 | 441.0 | 289.0 | Neg | *y*=-0.0142216+1.06768*×* | 0.997 | 1.63 | 1.28 | 0.1-5 | 19.5/28.5 | 0.9984 | 0.9946 | 0.0229 | 3 |
| 23 | Piceid | 17.2 | 391.0 | 135/106.9 | Poz | *y*=0.00772525+25.4181*×* | 0.999 | 1.94 | 1.16 | 0.05-2.5 | 13.8/17.8 | 1.0042 | 0.9979 | 0.0199 | 1 |
| 24 | *p*-Coumaric acid | 17.8 | 163.0 | 93.0 | Neg | *y*=0.0249034+18.5180*×* | 0.999 | 1.92 | 1.43 | 0.1-5 | 25.9/34.9 | 1.0049 | 1.0001 | 0.0194 | 1 |
| 25 | Ferulic acid-D3-IS*^h^* | 18.8 | 196.2 | 152.1 | Neg | N.A. | N.A. | N.A. | N.A. | N.A. | N.A. | N.A. | N.A. | 0.0170 | 1 |
| 26 | Ferulic acid | 18.8 | 192.8 | 149.0 | Neg | *y*=-0.0735254+1.34476*×* | 0.999 | 1.44 | 0.53 | 1-50 | 11.8/15.6 | 0.9951 | 0.9976 | 0.0181 | 1 |
| 27 | Sinapic acid | 18.9 | 222.8 | 193.0 | Neg | *y*=-0.0929932+0.836324*×* | 0.999 | 1.45 | 0.52 | 0.2-10 | 65.2/82.3 | 1.0031 | 1.0037 | 0.0317 | 1 |
| 28 | Coumarin | 20.9 | 146.9 | 103.1 | Poz | *y*=0.0633397+136.508*×* | 0.999 | 2.11 | 1.54 | 0.05-2.5 | 214.2/247.3 | 0.9950 | 0.9958 | 0.0383 | 1 |

*^a^*R.T.: Retention time, *^b^*MI (*m/z):* Molecular ions of the standard analytes (m/z ratio), *^c^*FI (*m/z):* Fragment ions *^d^r*^2^: Coefficient of determination, *^e^RSD*: Relative standard deviation, *^f^LOD*/*LOQ* (µg/L): Limit of detection/quantification, *^g^U* (%): percent relative uncertainty at 95% confidence level (*k* = 2), *^h^*IS: Internal standard, *^i^*Gr. No: Represents grouping of internal standards, these numbers indicate which IS stands for which phenolic compound.

**Table 2.** Analytical method validation parameters that belong to the LC-MS/MS method (Continued)

| No | Analytes | RT*^a^* | M.I. (m/z)*^b^* | F.I. (m/z)*^c^* | Ion. mode | Equation | *r^2d^* | *RSD*%*^e^* | | Linearity Range (mg/L) | *LOD*/*LOQ* (µg/L)*^f^* | Recovery (%) | | *U^g^* | Gr. No |
| --- | --- | --- | --- | --- | --- | --- | --- | --- | --- | --- | --- | --- | --- | --- | --- |
|  |  |  |  |  |  |  |  | Interday | Intraday |  |  | Interday | Intraday |  |  |
| 29 | Salicylic acid | 21.8 | 137.2 | 65.0 | Neg | *y*=0.239287+153.659*×* | 0.999 | 1.48 | 1.18 | 0.05-2.5 | 6.0/8.3 | 0.9950 | 0.9998 | 0.0158 | 1 |
| 30 | Cynaroside | 23.7 | 447.0 | 284.0 | Neg | *y*=0.280246+6.13360*×* | 0.997 | 1.56 | 1.12 | 0.05-2.5 | 12.1/16.0 | 1.0072 | 1.0002 | 0.0366 | 2 |
| 31 | Miquelianin | 24.1 | 477.0 | 150.9 | Neg | *y*=-0.00991585+5.50334*×* | 0.999 | 1.31 | 0.95 | 0.1-5 | 10.6/14.7 | 0.9934 | 0.9965 | 0.0220 | 2 |
| 32 | Rutin-D3-IS*^h^* | 25.5 | 612.2 | 304.1 | Neg | N.A. | N.A. | N.A. | N.A. | N.A. | N.A. | N.A. | N.A. | N.A. | 2 |
| 33 | Rutin | 25.6 | 608.9 | 301.0 | Neg | *y*=-0.0771907+2.89868*×* | 0.999 | 1.38 | 1.09 | 0.1-5 | 15.7/22.7 | 0.9977 | 1.0033 | 0.0247 | 2 |
| 34 | isoquercitrin | 25.6 | 463.0 | 271.0 | Neg | *y*=-0.111120+4.10546*×* | 0.998 | 2.13 | 0.78 | 0.1-5 | 8.7/13.5 | 1.0057 | 0.9963 | 0.0220 | 2 |
| 35 | Hesperidin | 25.8 | 611.2 | 449.0 | Poz | *y*=0.139055+13.2785*×* | 0.999 | 1.84 | 1.35 | 0.1-5 | 19.0/26.0 | 0.9967 | 1.0043 | 0.0335 | 2 |
| 36 | *o*-Coumaric acid | 26.1 | 162.8 | 93.0 | Neg | *y*=0.00837193+11.2147*×* | 0.999 | 2.11 | 1.46 | 0.1-5 | 31.8/40.4 | 1.0044 | 0.9986 | 0.0147 | 1 |
| 37 | Genistin | 26.3 | 431.0 | 239.0 | Neg | *y*=1.65808+7.57459*×* | 0.991 | 2.01 | 1.28 | 0.1-5 | 14.9/21.7 | 1.0062 | 1.0047 | 0.0083 | 2 |
| 38 | Rosmarinic acid | 26.6 | 359.0 | 197.0 | Neg | *y*=-0.0117238+8.04377*×* | 0.999 | 1.24 | 0.86 | 0.1-5 | 16.2/21.2 | 1.0056 | 1.0002 | 0.0130 | 1 |
| 39 | Ellagic acid | 27.6 | 301.0 | 284.0 | Neg | *y*=0.00877034+0.663741*×* | 0.999 | 1.57 | 1.23 | 0.4-20 | 56.9/71.0 | 1.0005 | 1.0048 | 0.0364 | 1 |
| 40 | Cosmosiin | 28.2 | 431.0 | 269.0 | Neg | *y*=-0.708662+8.62498*×* | 0.998 | 1.65 | 1.30 | 0.1-5 | 6.3/9.2 | 0.9940 | 0.9973 | 0.0083 | 2 |
| 41 | Quercitrin | 29.8 | 447.0 | 301.0 | Neg | *y*=-0.00153274+3.20368*×* | 0.999 | 2.24 | 1.16 | 0.1-5 | 4.8/6.4 | 0.9960 | 0.9978 | 0.0268 | 2 |
| 42 | Astragalin | 30.4 | 447.0 | 255.0 | Neg | *y*=0.00825333+3.51189*×* | 0.999 | 2.08 | 1.72 | 0.1-5 | 6.6/8.2 | 0.9968 | 0.9957 | 0.0114 | 2 |
| 43 | Nicotiflorin | 30.6 | 592.9 | 255.0/284.0 | Neg | *y*=0.00499333+2.62351*×* | 0.999 | 1.48 | 1.23 | 0.05-2.5 | 11.9/16.7 | 0.9954 | 1.0044 | 0.0108 | 2 |
| 44 | Fisetin | 30.6 | 285.0 | 163.0 | Neg | *y*=0.0365705+8.09472*×* | 0.999 | 1.75 | 1.19 | 0.1-5 | 10.1/12.7 | 0.9980 | 1.0042 | 0.0231 | 3 |
| 45 | Daidzein | 34.0 | 253.0 | 223.0 | Neg | *y*=-0.0329252+6.23004*×* | 0.999 | 2.18 | 1.73 | 0.1-5 | 9.8/11.6 | 0.9926 | 0.9963 | 0.0370 | 3 |
| 46 | Quercetin-D3-IS*^h^* | 35.6 | 304.0 | 275.9 | Neg | N.A. | N.A. | N.A. | N.A. | N.A. | N.A. | N.A. | N.A. | N.A. | 3 |
| 47 | Quercetin | 35.7 | 301.0 | 272.9 | Neg | *y*=+0.00597342+3.39417*×* | 0.999 | 1.89 | 1.38 | 0.1-5 | 15.5/19.0 | 0.9967 | 0.9971 | 0.0175 | 3 |
| 48 | Naringenin | 35.9 | 270.9 | 119.0 | Neg | *y*=-0.00393403+14.6424*×* | 0.999 | 2.34 | 1.69 | 0.1-5 | 2.6/3.9 | 1.0062 | 1.0020 | 0.0392 | 3 |
| 49 | Hesperetin | 36.7 | 301.0 | 136.0/286.0 | Neg | *y*=+0.0442350+6.07160*×* | 0.999 | 2.47 | 2.13 | 0.1-5 | 7.1/9.1 | 0.9998 | 0.9963 | 0.0321 | 3 |
| 50 | Luteolin | 36.7 | 284.8 | 151.0/175.0 | Neg | *y*=-0.0541723+30.7422*×* | 0.999 | 1.67 | 1.28 | 0.05-2.5 | 2.6/4.1 | 0.9952 | 1.0029 | 0.0313 | 3 |
| 51 | Genistein | 36.9 | 269.0 | 135.0 | Neg | *y*=-0.00507501+12.1933*×* | 0.999 | 1.48 | 1.19 | 0.05-2.5 | 3.7/5.3 | 1.0069 | 1.0012 | 0.0337 | 3 |
| 52 | Kaempferol | 37.9 | 285.0 | 239.0 | Neg | *y*=-0.00459557+3.13754*×* | 0.999 | 1.49 | 1.26 | 0.05-2.5 | 10.2/15.4 | 0.9992 | 0.9990 | 0.0212 | 3 |
| 53 | Apigenin | 38.2 | 268.8 | 151.0/149.0 | Neg | *y*=0.119018+34.8730*×* | 0.998 | 1.17 | 0.96 | 0.05-2.5 | 1.3/2.0 | 0.9985 | 1.0003 | 0.0178 | 3 |
| 54 | Amentoflavone | 39.7 | 537.0 | 417.0 | Neg | *y*=0.727280+33.3658*×* | 0.992 | 1.35 | 1.12 | 0.05-2.5 | 2.8/5.1 | 0.9991 | 1.0044 | 0.0340 | 3 |
| 55 | Chrysin | 40.5 | 252.8 | 145.0/119.0 | Neg | *y*=-0.0777300+18.8873*×* | 0.999 | 1.46 | 1.21 | 0.05-2.5 | 1.5/2.8 | 0.9922 | 1.0050 | 0.0323 | 3 |
| 56 | Acacetin | 40.7 | 283.0 | 239.0 | Neg | *y*=-0.559818+163.062*×* | 0.997 | 1.67 | 1.28 | 0.02-1 | 1.5/2.5 | 0.9949 | 1.0011 | 0.0363 | 3 |

*^a^*R.T.: Retention time, *^b^*MI (*m/z):* Molecular ions of the standard analytes (m/z ratio), *^c^*FI (*m/z):* Fragment ions *^d^r^2^*: Coefficient of determination, *^e^RSD*: Relative standard deviation, *^f^LOD*/*LOQ* (µg/L): Limit of detection/quantification, *^g^U* (%): percent relative uncertainty at 95% confidence level (*k* = 2), *^h^*IS: Internal standard, *^i^*Gr. No: Represents grouping of internal standards, these numbers indicate which IS stands for which phenolic compound.

**Mass spectrometer and chromatograph conditions for LC-MS/MS analysis**

The reversed-phase UHPLC was equipped with an autosampler (SIL-30AC model), a column oven (CTO-10ASvp model), binary pumps (LC-30AD model), and a degasser (DGU- 20A3R model). The chromatographic conditions were optimized in order to achive optimum separation for 53 phytochemicals and overcome the suppression effects. The chromatographic seperation was performed on a reversed phase Agilent Poroshell 120 EC-C18 model (150 mm×2.1 mm, 2.7 µm) analytical column. The column temperature was set to 40°C. The elution gradient was composed of eluent A (water+5 mM ammonium formate+0.1% formic acid) and eluent B (methanol+5 mM ammonium formate+0.1% formic acid). The following gradient elution profile was used: 20-100% B (0-25 min), 100% B (25-35 min), 20% B (35-45 min). Furthermore, the solvent flow rate and injection volume were settled as 0.5 mL/min and 5 µL, respectively.

The mass spectrometric detection was carried out using a Shimadzu LCMS-8040 model tandem mass spectrometer equipped with an electrospray ionization (ESI) source operating in both negative and positive ionization modes. LC-ESI-MS/MS data were acquired and processed by LabSolutions software (Shimadzu). The MRM (multiple reaction monitoring) mode was used for the quantification of the phytochemicals. The MRM metot was optimized to selectively detect and quantify phytochemical compounds based on the screening of specified precursor phytochemical-to-fragment ion transitions. The collision energies (CE) were optimized in order to generate optimal phtochemical fragmentation and maximal transmission of the desired product ions. The MS operatinging conditions were applied as: drying gas (N2) ﬂow, 15 L/min; nebulizing gas (N_2_) flow, 3 L/min; DL temperature, 250°C; heat block temperature, 400°C, and interface temperature, 350°C.


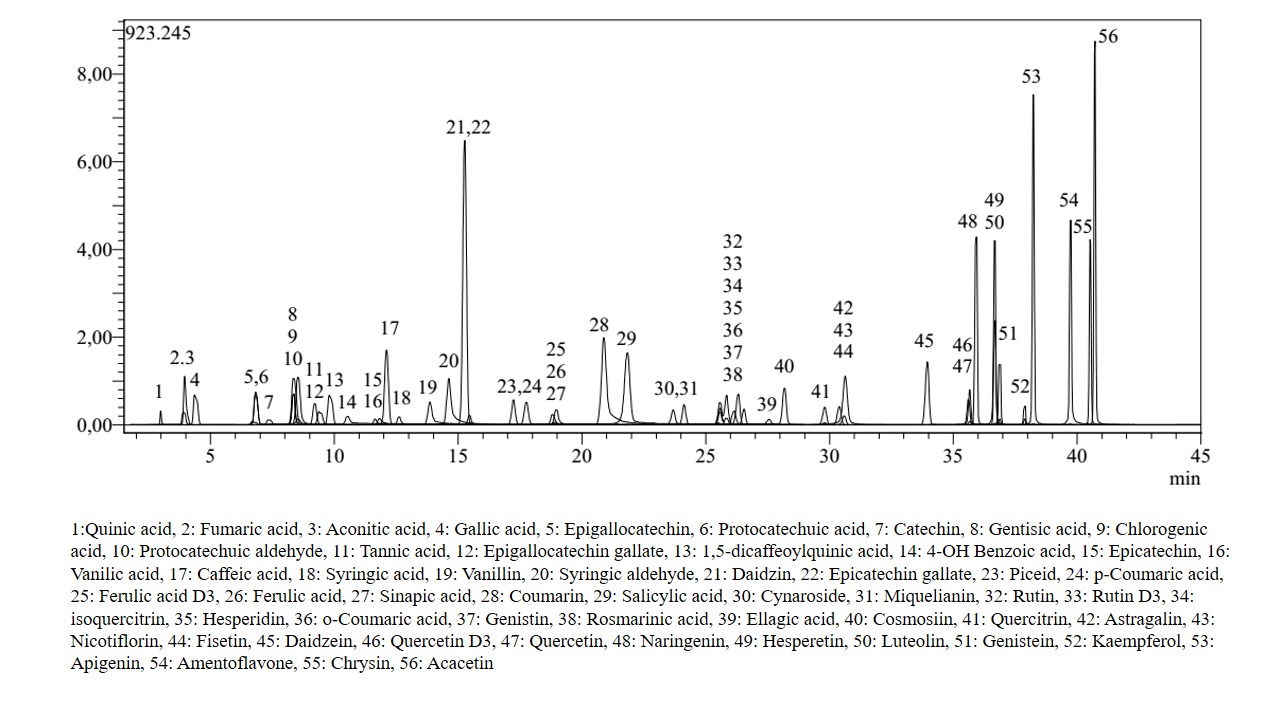


**Fig S9.** TIC (Total Ion Chromatogram) chromatogram of standard phenolic compounds
